# Oral Delivery of Double-Stranded RNA in Larvae of the Yellow Fever Mosquito, *Aedes aegypti:* Implications for Pest Mosquito Control

**DOI:** 10.1673/031.013.6901

**Published:** 2013-07-15

**Authors:** Aditi D. Singh, Sylvia Wong, Calen P. Ryan, Steven Whyard

**Affiliations:** Department of Biological Sciences, University of Manitoba, Winnipeg, Manitoba, Canada

**Keywords:** β-tubulin, chitin synthase, heat shock protein 83, insecticides, RNA interference

## Abstract

RNA interference has already proven itself to be a highly versatile molecular biology tool for understanding gene function in a limited number of insect species, but its widespread use in other species will be dependent on the development of easier methods of double-stranded RNA (dsRNA) delivery. This study demonstrates that RNA interference can be induced in the mosquito *Aedes aegypti* L. (Diptera: Culicidae) simply by soaking larvae in a solution of dsRNA for two hours. The mRNA transcripts for *β-tubulin, chitin synthase-1* and -2, and *heat shock protein 83* were reduced between 30 and 50% three days post-dsRNA treatment. The dsRNA was mixed with a visible dye to identify those individuals that fed on the dsRNA, and based on an absence of RNA interference in those individuals that contained no dye within their guts, the primary route of entry of dsRNA is likely through the gut epithelium. RNA interference was systemic in the insects, inducing measurable knock down of gene expression in tissues beyond the gut. Silencing of the *β-tubulin* and *chitin synthase-1* genes resulted in reduced growth and/or mortality of the larvae, demonstrating the utility of dsRNA as a potential mosquito larvicide. Silencing of *chitin synthase-2* did not induce mortality in the larvae, and silencing of *heat shock protein 83* only induced mortality in the insects if they were subsequently subjected to a heat stress. *Drosophila melanogaster* Meigen (Diptera: Drosophilidae) larvae were also soaked in dsRNA designed to specifically target either their *own β-tubulin* gene, or that of *A. aegypti*, and significant mortality was only seen in larvae treated with dsRNA targeting their own gene, which suggests that dsRNA pesticides could be designed to be species-limited.

## Introduction

RNA interference (RNAi) is a double-stranded RNA (dsRNA)-mediated mechanism of silencing gene expression in eukaryotes. Following delivery into a cell, dsRNA is cleaved by Dicer into short (∼21 nt) interfering RNAs. The short interfering RNAs are then incorporated into an RNA-induced silencing complex, which facilitates binding to and cleaving of complementary mRNA sequences, thereby preventing translation of the gene (reviewed in Hannon 2002; Kim and Rossi 2008). The basic components of the RNAi machinery are found in essentially all eukaryotes, and hence, RNAi is now widely exploited as a reverse genetics tool to assess gene functions in a broad range of species. The application of RNAi in most species under study is only limited by how easily the dsRNA can be delivered to the target cells.

In studies involving insects, direct injections of *in vitro*-synthesized dsRNA into virtually any developmental stage can produce loss-of-function mutants (Misquitta and Paterson 1999; Bettencourt et al. 2002; Beye et al. 2002; Bucher et al. 2002; Rajagopal et al. 2002; Amdam et al. 2003; Gatehouse et al. 2004; Tomoyasu and Denell 2004). While haemocoel injection of dsRNA is still the most common method of dsRNA delivery forinsects, it is a rather laborious technique and numerous insects may not survive the injection process. A simpler method of dsRNA delivery that is less injurious is delivery through the diet. Oral delivery by droplet or liquid feeding (Turner et al. 2006; Bautista et al. 2009; Maori et al. 2009; Whyard et al. 2009; Li et al. 201 la) or adding dsRNA to dry diets (Shakesby et al. 2009; Whyard et al. 2009; Li et al. 2011b) has been successful in a small, but growing number of species. For some insects, ingested dsRNA fails to induce RNAi (Rajagopal et al. 2002; Terenius et al. 2011), but in three studies examining RNAi in dipterans, oral delivery was facilitated by using either a lipid-based transfection reagent (Whyard et al. 2009; Cancino-Rodezno et al. 2010) or chitosan nanoparticles to stabilize the dsRNAs (Zhang et al. 2010).

Feeding dsRNAs to insects has not only provided researchers with an excellent molecular biology tool to assess gene function, but it also has great potential for pest insect control. Two research groups demonstrated that plants could be genetically engineered to express insect-specific dsRNAs that would kill the insects that feed on them (Baum et al. 2007; Mao et al. 2007). Given that RNAi operates in a very sequence-specific manner, it may be possible to develop species-specific dsRNA pesticides. Even when a highly conserved gene such as β-tubulin is targeted by RNAi,dsRNAs specific to the unique 3′ untranslated region of the mRNAs could selectively kill only one species of *Drosophila* without adversely affecting other closely related drosophilids (Whyard et al. 2009).

Much of the interest in RNAi-based control of pest insects has focussed on crop pests, using transgenic plants that express insecticidal dsRNAs (Gordon and Waterhouse 2007; Price and Gatehouse 2008; Huvenne and Smagghe 2010; Jagtap et al. 2011). Controlling medically-important, disease vectoring pests using RNAi has not attracted as many proponents, due in large part to the unique challenges of delivering dsRNA to these pests. Mosquitoes unarguably represent the most serious disease vectors, infecting millions of people with life-threatening illnesses such as malaria and dengue each year (World Health Organization 2009, 2010). Chemical pesticides are used extensively to control many mosquitoes, but with growing concerns about the increasing frequency of insecticide resistance (Hemingway et al. 2002) and the negative impacts of current pesticides on non-target species (reviewed in Paoletti and Pimentai 2000; Nicholson 2007), it is important that we continue to search for new, and ideally more species-selective pesticides to control these serious pests. The prospect of using RNAi to control mosquitoes is intriguing, but will require significant improvements in dsRNA delivery, as well as higher throughput analyses to identify appropriate dsRNA targets.

To date, RNAi in mosquitoes has been used primarily as a molecular biology tool to identify the roles of genes relevant to their development (Attardo et al. 2003; Hoa et al. 2003; Roy et al. 2007; Smith and Linser 2009; Clemons et al. 2010; Kim et al. 2010) and disease potential (Adelman et al. 2002; Caplen et al. 2002; Zhu et al. 2003; Boisson et al. 2006; Franz et al. 2006; Scott et al. 2010; Wu et al. 2010). All of these studies have involved delivery of dsRNA to adult mosquitoes using microinjection or using cell cultures rather than whole organisms. Recently however, it was shown that *Aedes aegypti* L. (Diptera: Culicidae) could be fed dsRNAs bound by the transfection reagent Effectene (Qiagen, www.qiagen.com) to cause knockdown of the MAPK p38 gene, increasing the insects’ susceptibility to Cry toxins (Cancino-Rodezno et al. 2010). Similarly, dsRNAs bound to chitosan nanoparticles fed to larvae of the malaria mosquito, *Anopheles gambiae*, caused significant knockdown of two chitin synthase genes. While the dsRNAs were not insecticidal, chitin formation was reduced sufficiently to increase the susceptibility of the larvae to diflubenzuron, calcofluor white, and dithiothreitol (Zhang et al. 2010).

This study demonstrates the insecticidal action of orally-delivered dsRNAs in larvae of the yellow fever mosquito, *A. aegypti*. In contrast to the studies by Cancino-Rodezno et al. (2010) and Zhang et al. (2010), in our study it was found that a relatively brief soaking in dsRNA, without the use of transfection reagents or dsRNA carriers, was sufficient to induce RNAi, and can either stunt growth or kill mosquito larvae. While none of the 3 targeted genes (*β-tubulin, chitin synthase-1*, and *heat shock protein 83*) are unique to mosquitoes, their RNAi-induced effects serve as a proof-of-principle that a variety of genes may serve as possible targets for dsRNA-based pesticides for mosquito control.

## Materials and Methods

### DsRNA preparation

Total RNA was extracted from 10 larval *A. aegypti*, using QIAshredders (Qiagen) to homogenize tissues and an RNeasy RNA extraction kit (Qiagen). RNA was treated with amplification grade DNase I (Invitrogen, www.invitrogen.com) and 1 μg was used to synthesize cDNA using a First Strand cDNA Synthesis kit (Invitrogen).

Two *A. aegypti* chitin synthase genes, sharing 50% identity at the predicted amino acid level, were identified by BLAST comparisons to the annotated *Anopheles gambiae AgCHS1* (GenBank accession no. XM_321336) and *AgCHS2* (GenBank accession no. AY056833) cDNA sequences (Zhang et al. 2010), and hereafter are referred to as *AeCS1* and *AeCS2* (previously described by Kato et al. 2006), respectively. Fragments of the *β-tubulin* (*βtub), AeCS1, AeCS2*, and *heat shock protein 83 (hsp83*) genes were PCR-amplified from the cDNA using the primers listed in Table 1. To avoid cross-silencing other genes, each dsRNA target sequence was screened for cross-homologies within the *A. aegypti* genome using BLAST analyses to ensure that there were no shared identities greater than 19 nucleotides in length. The gene fragments were subcloned into the cloning vector pDrive (Qiagen), and later excised from pDrive using either *ApaI* and *PstI* or *MluI* and *NotI* restriction enzymes, then ligated into a similarly-digested plasmid pL4440, a vector possessing convergent T7 promoters (kindly provided by Andrew Fire, Stanford University). The *β-glucuronidase* (*gus*) gene, a bacterial gene specific to *Escherichia coli*, was amplified by PCR from the pBacPAK8-GUS plasmid (Clontech, www.clontech.com) using the following primers:

GusF 5′ TGGTCCGTCCTGTAGAAACC

GusR 5′ CCCCACCGAGGCTGTAGC

The 1.87 kb PCR product was cloned into the dsRNA transcription plasmid pL4440, as described above, to be used as a negative control.

DNA templates for *in vitro* transcription of each of the gene fragments in pL4440 were PCR-amplified using the following pL4440-specific primers:

pL4440F 5′ ACCTGGCTTATCGAA

pL4440R 5′ TAAAACGACGGCCAGT

PCR products were then purified using a QIAquick PCR purification kit (Qiagen). The MEGAscript RNAi kit (Ambion, www.invitrogen.com/ambion) was then used for *in vitro* transcription and purification of dsRNAs.

### Bioassays

*A. aegypti* were reared at 25° C, 50% humidity, on a 16:8 L:D photoperiod. Females were fed warmed rat blood encased in stretched Nescofilm (Karlan Research Products, www.karlan.com). Mosquito eggs were allowed to develop for a minimum of one week, then were submerged in dechlorinated tap water to induce hatching. Larvae were maintained on a ground liver powder and guinea pig chow diet.

Larvae were initially soaked in solutions of dsRNA containing a visible dye to assess the extent of ingestion of the dsRNA solution. Groups of 20 first instar larvae were soaked for 0, 1, and 2 hr in 75 μl water containing 0.5 μg/ μl *β-tub*-dsRNA and 0.5% bromophenol blue. The larvae were photographed using a Zeiss Photomicroscope III (www.zeiss.com) equipped with a Sony DXC-390 color video camera (www.sony.com), and the intensity of the dye in the gut was calculated using ImageJ image processing software (http://rsbweb.nih.gov/ij/). The extent of dye in the gut was correlated with the extent of knockdown of *the β-tub* gene expression using quantitative reverse transcriptase PCR (see section below). Once it was determined that dsRNA was being ingested by larvae, subsequent dsRNA treatments were performed without the addition of the dye.

First instar larvae (< 24 hr old) were then treated in groups of 50 in a final volume 75 μl of dsRNA at various concentrations (ranging from 0.02 to 0.5μg/μl for β*-tub*-dsRNA, or 0.5 μg/μl for *AeCS1-, AeCS2-* and *hsp83-*dsRNAs) in a 2 mL microfuge tube. Negative control larvae were treated with either water alone or with the *E. coli*-specific *gus*-dsRNA, which has no homology with any mosquito genes and has had no adverse effects on several other insects (Whyard et al. 2009). As lipid-based transfection reagents may facilitate dsRNA uptake in some insects (Whyard et al. 2009), parallel bioassay experiments were also conducted using dsRNA that had first been mixed with Lipofectamine 2000 (Invitrogen), using the protocol described by the manufacturer. Larvae were soaked in the dsRNA solutions for 2 hr at 21° C, and then transferred to 12-well tissue culture plates, which were also maintained at 21° C, and provided 5 mg/mL lab rat diet (Purina Mills, www.purinamills.com) suspended in water as a source of food on a daily basis. This amount of food was equivalent to half-rations; 10 mg/mL of food per day typically enabled the insects to develop to the pupal stage in 5 days. The lower rearing temperature and reduced food during these bioassays slowed their development and facilitated easier monitoring of differential growth rates and/or survivorship. Growth and/or survival of the larvae were observed over a 2-week period, by which time all non-treated larvae had pupated and developed into adults. Larvae treated with *hsp83-*dsRNA were subjected to a 2 hr heat shock at 37° C, 2 days post-dsRNA treatment, then returned to 21° C for rearing to assess whether they were more sensitive to a heat stress than *gus*-dsRNA treated controls.

To assess whether the RNAi effect had spread beyond gut tissues, first instar larvae were exposed to *β-tub*-dsRNA as described above, and 3 days after the initial 2 hr soaking in 0.5 μl/μg concentrations of dsRNA the larvae were dissected to separate guts from the remaining carcass. The tissues were pooled in groups of 25 and stored in *RNAlater* (Ambion) at -80° C until the extent of RNAi could be determined (described below).

### Quantitative RT-PCR to measure gene knockdown

Ten to 20 larvae from each treatment, or 25 guts and/or carcasses, were collected and pooled together 3 days after the single 2 hr dsRNA soakings. RNA extractions and cDNA syntheses were performed as above. Only live insects were used for the RNA extractions, as the RNA in dead insects could have degraded. The cDNA from each replicate treatment was then used to assess the extent of RNAi by measuring levels of gene expression using qRT-PCR. Reactions were performed in triplicate on a BioRad iQ5 Real-Time PCR Detection System (www.bio-rad.com) using the primers listed in Table 2. S7 ribosomal protein (*S7rp*) gene expression was used as an internal reference to compare levels of RNAi. A single reference gene was deemed sufficient, as the PCR efficiencies of the primer sets were calculated using the method of Pfaffl (2001), and were found to be essentially equivalent for all genes targeted by RNAi (*βtub, AeCS1, AeCS2*, and *hsp83*) and for the *S7rp* reference gene, with values ranging between 95.2 and 98.1%. Melt curve analyses were also performed and confirmed that only a single product was amplified with each primer pair in every sample. Analysis of gene expression was performed using the 2^-ΔΔC^Tmethod (Livak and Schmittgen 2001), comparing expression in specific dsRNA treated samples to *gus*-dsRNA treated samples.

**Figure 1.**
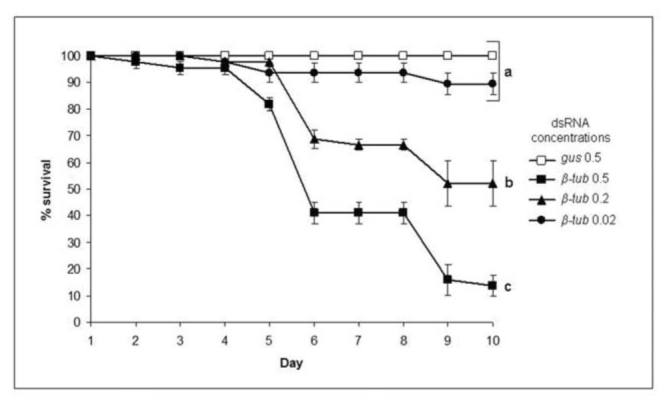
Survival of *Aedes aegypti* larvae after soaking in various concentrations of *β-tubulin*-dsRNA. Values represent the means and standard errors from 3 replicates. Different letters (a, b, and c) indicate significantly different survival rates from other treatments (ANOVA, *p* < 0.05) after 10 days development. Note that the survival rates for all *gus*-dsRNA doses were not significantly different from one another, and only the highest dose is displayed. High quality figures are available online.

### Delivery of *A. aegypti* dsRNA to *Drosophila*

*Drosophila melanogaster* Meigen (Diptera: Drosophilidae) (Oregon R strain) cultures were reared on an agar-yeast-cornmeal medium at room temperature on a 12:12 L:D photoperiod. RNA was extracted from 50 *D. melanogaster* first instar larvae, and cDNA was prepared as previously described. A 128 bp fragment of the *D. melanogaster βTub56D* gene (*Dmtub*) was amplified using the primers listed in Table 1, cloned into pL4440, and dsRNA was synthesized as described above. A 328 bp fragment of the *A. aegypti β-tub* gene (*Aetub*), homologous to *Dmtub*, was also amplified, cloned, and used for dsRNA synthesis as described above. These two gene fragments did not contain any 19–21 identical nucleotide lengths (results not shown).

The *Aetub-* and the *Dmtub*-dsRNAs were fed to newly hatched *D. melanogaster* first instar larvae by soaking them in 0.5 μg/μl solutionsof dsRNA encapsulated in Lipofectamine 2000 (Invitrogen) for 2 hr. Larvae were also exposed to *gus*-dsRNA, which served as the negative control. After treatment, the larvae were placed in Petri dishes containing a thin layer of agar-yeast-cornmeal medium for further development, and were monitored for mortality. Subsets of treated larvae were collected for RNA extractions to assess the extent of RNAi of the *β-tubulin* transcripts using qRT-PCR as described above, using the primers listed in Table 2. The ribosomal protein L32 (*RpL32*) gene was used as an internal reference gene for assessing RNAi in *D. melanogaster*.

## Results

### Soaking in β-tub-dsRNA induces RNAi and kills *A. aegypti* larvae

Soaking first instar mosquito larvae in a solution of *β-tub*-dsRNA for a single 2 hr period was sufficient to induce mortality in a dosedependent manner (Figure 1). β-tubulin is an essential component of a cell's cytoskeleton, and loss-of-function mutations of the *β-tub* gene are lethal in various organisms (Skop et al. 2004; Sonnichsen et al. 2005; Baum et al. 2007; Buszczak et al. 2007). Reduction of *βtub* expression by RNAi in the mosquitoes presumably adversely affected the gut cell's normal functions, which resulted in failure to acquire sufficient nutrients and death. To determine whether the mosquito larvae were ingesting the dsRNA, initial trials were performed with bromophenol blue added to *βtub*-dsRNA solutions. Digital analysis of photographs of the larvae revealed that after a 2 hr exposure, the guts of the larvae were filled with dye, whereas following shorter exposure periods, considerably less and highly variable amounts of dye had been ingested (Table 3). No staining of any other tissues was observed. QRT-PCR analyses of these larvae 3 dayspost-treatment confirmed that those individuals exposed to the dsRNA solution for 2 hr had both high levels of dye within their guts and showed a 42% reduction in *β-tub* transcripts relative to the negative controls (Table 3). A small number of larvae were found with no observable dye within their guts after the 2 hr exposure, and they showed no significant reduction in *β-tub* transcripts relative to the negative controls (n = 10, two-tailed Student t-test, *p* = 0.55). Based on these observations, it is likely that in this dsRNA soaking treatment, the gut was the primary route of entry of the dsRNA.

Lipid- (Whyard et al. 2009; Cancino-Rodezno et al. 2010) or polyamine-based (Zhang et al. 2010) transfection reagents have been used to improve dsRNA delivery to some insects. As dipterans are thought to lack the dsRNA transporter SID-1 (Gordon and Waterhouse 2007), the use of liposomes or other transfection reagents may improve delivery of dsRNA to the gut cells. Somewhat surprisingly, in our study, encapsulation of the dsRNA in Lipofectamine 2000 did not affect the dsRNA-induced mortality of mosquito larvae in these feeding assays (Table 4). For that reason, all subsequent dsRNA treatments were conducted without the use of liposomes.

Exposures of the mosquito larvae to the *gus*-dsRNA and the lowest *β-tub*-dsRNA concentration (0.02 μg/μl) had no significant impact on larval survival relative to the negative controls treated with no dsRNA (Figure 1). However, when larvae were soaked in the two higher dsRNA concentrations of *β-tub*-dsRNA (0.2 and 0.5 μg/μl), significant impacts on survival were observed, with only 52.1 ± 8.3 and 13.7 ± 3.9% survival, respectively, 9 days post-treatment (Figure 1).

### Soaking in *AeCS1-* and *hsp83*-dsRNAs stunts growth or kills *A. aegypti* larvae

**Figure 2.**
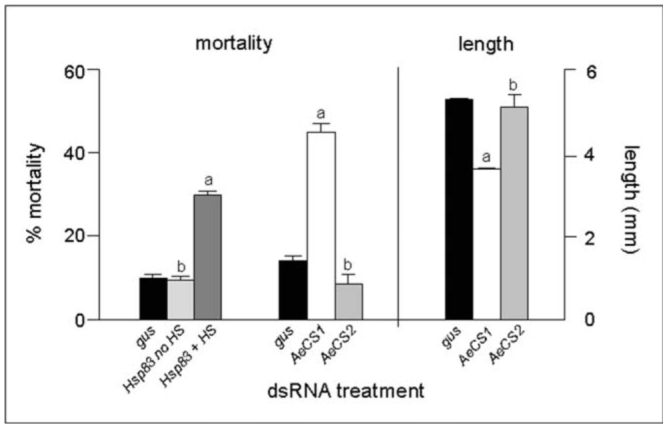
Mortality and growth of Aedes *aegypti* larvae 7 days post-treatment with *AeCS1-, AeCS2-, and hsp83*-dsRNA as day-old larvae, compared to *gus*-dsRNA treated controls. For larvae treated with *hsp83*-dsRNA, some larvae were exposed to no heat shock (no HS) while others were subjected to a 2 hr heat shock (+ HS). Values represent the means and standard errors from 3 replicates. High quality figures are available online.

To determine if other genes could also be targeted by ingestion of dsRNA, mosquito larvae were treated with 0.5 μg/μl of dsRNAs specific to two chitin synthase genes (*AeCS1* and *AeCS2*) and the putative chaperone protein (*hsp83*). In insects, chitin synthase-1 is a key enzyme involved in the synthesis of chitin of the insect's exoskeleton, whereas chitin synthase-2 is involved in synthesis of chitin associated with the peritrophic membrane within the midgut (Arakane et al. 2005; Merzendorfer 2006). The malaria mosquito, *An. gambiae*, similarly expresses chitin synthase 1 (*AgCHS1*) in the cuticle of larvae and not in the midgut, whereas chitin synthase 2 (*AgCHS2*) is restricted to the midgut (Zhang et al. 2010). The two *A. aegypti* chitin synthase proteins, *AeCS1 and AeCS2*, shared 91% and 83% identity, respectively, with the corresponding proteins identified in *An. gambiae*. Using qRT-PCR analyses on cDNAs derived from isolated midguts and remaining carcasses, *AeCS1* was found to be expressed predominantly in the carcass and not in the midgut, while *AeCS2* was detectable only in the midgut (Table 5). *A. aegypti* larvae treated with dsRNA targeting *AeCS1* showed both decreased growth and increased mortality over time, relative to larvae treated with the control *gus*-dsRNA (Figure 2). Larval mortality, assessed 7 days post-treatment, was approximately 3 times higher in insects treated with 0.5 μg/μl *AeCS1*-dsRNA, relative to the *gus*-dsRNA-treated controls. Of the insects that did not die following the *AeCS1*-dsRNA treatment, many showed stunted growth, being nearly 1/3 shorter than the negative control larvae 1 week post-treatment (Figure 2). None of these stunted larvae developed into adults over a 2-week period, whereas the majority (> 90%) of negative control insects eclosed before the 2-week period elapsed.

Interestingly, larvae treated with *AeCS2*-dsRNA showed no evidence of stunting and developed at the same rate as the *gus*-dsRNA controls. Measurements of gene expression 3 days post-treatment, using qRT-PCR, determined that *AeCS1* expression, assessed from whole body RNA extractions, was reduced almost 40%, while *AeCS2* expression was reduced 54%, relative to the *gus*-dsRNA negative controls (Table 5). Even though gene expression was not fully knocked down, the reduction of *AeCS1* transcripts was sufficient to adversely affect the survival of almost half of the insects treated with this dsRNA (Figure 2). This result is not surprising, as loss-of-function mutant alleles of *CS-1* in *D. melanogaster* are also lethal (McQuilton et al. 2012). The lack of any adverse effect on growth and development of *AeCS2*-dsRNA-treated larvae suggests that either the peritrophic membrane was not sufficiently disrupted by RNAi or that disruption of the peritrophic membrane alone was not sufficient to kill the *A. aegypti* larvae.

*Hsp83* encodes a heat shock protein that aids in folding and unfolding proteins under stress (Xiao and Lis 1989). In the mosquito *A. aegypti, hsp83* expression increases several fold when the insects are subjected to heat shock (Zhao et al. 2010). In other insect species, including the African migratory locust, *Locusta migratoria* (Whyard et al. 1986), the light brown applemoth, *Epiphyas postvittana* (Lester and Greenwood 1997), the flesh fly, *Sarcophaga crassipalpis* (Chen et al. 1991), as well as the model insect, *D. melanogaster* (Krebs and Feder 1998), pre-treatment at a sublethal heat shock temperature can induce production of heat shock proteins, which can subsequently confer protection to the insects if later subjected to a brief lethal temperature.

Larvae that were treated with dsRNA *hsp83*-specific dsRNA, then heat shocked at 37° C, showed 3-fold greater mortality than control larvae (Figure 2), suggesting that reduction of *hsp83* transcripts leads to a reduced ability to tolerate a heat shock.

QRT-PCR analyses using cDNA derived from whole larvae 3 days post-treatment showed a statistically significant 36.2 ± 5.6% reduction of *hsp83* transcripts relative to the negative control gus-dsRNA treated larvae (Student *t*-test, *p* < 0.05). As was observed with the *AeCS1*-dsRNA treated larvae, this modest knockdown of *hsp83* transcripts was still sufficient to adversely affect the insects’ survival, reducing their ability to tolerate a brief heat shock.

### Soaking in dsRNA can induce systemic RNAi in *A. aegypti* larvae

**Figure 3.**
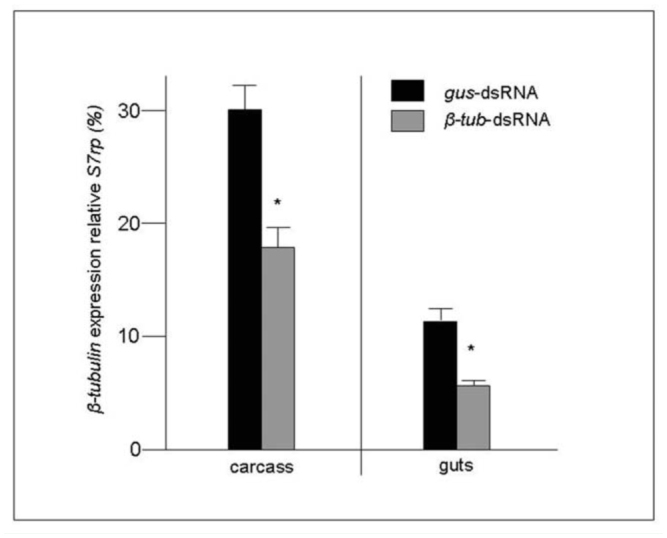
Relative *β-tubulin* expression, 3 days post-treatment, in guts and carcasses of dsRNA-treated day-old larvae. Gene expression values are relative to the reference gene, *S7rp*. Values represent the means and standard errors from 3 replicates. High quality figures are available online.

The initial experiments with dye mixed with the dsRNA solutions suggested that the gut was the main point of entry of the dsRNA when the larvae were soaked in a dsRNA solution. The knockdown of *AeCS1*, which was expressed in the epidermis beneath the exoskeleton, suggested that the dsRNA entering the gut can spread to other tissues. To assess the extent that dsRNA may spread beyond the gut tissues, *β-tub*-dsRNA-treated larvae were dissected to remove guts from the rest of the body (carcass) and qRT-PCR was used to assess the extent of RNAi in the two tissues. As expected, guts derived from β*-tub*-dsRNA-treated larvae showed a 50.1 ± 5.2% decrease in β*-tub* expression relative to *gus*-dsRNA-treated larvae (Figure 3). Interestingly, the carcass also showed a decrease in β*-tub* expression of 40.3 ± 6.0%, suggesting that following ingestion of the dsRNA, it can spread beyond the gut and affect other tissues.

### Effect of *A. aegypti* dsRNA on *D. melanogaster* larvae

To test the species-specificity of *A. aegypti* dsRNA targeting *β-tubulin*, first instar *D. melanogaster* larvae were soaked in *gus*-dsRNA, an 170 bp length dsRNA specific for the β*TubD56* gene of *D. melanogaster* (*Dmtub*-dsRNA), or a 328 bp length of dsRNA specific to the *A. aegypti β-tubulin* gene (*Aetub*-dsRNA) that had no 19 to 21 nucleotide lengths matching *βTubD56*.

The post-treatment mortalities of *D. melanogaster* larvae treated with 0.5 μg/μl of either *gus-* or *Aetub*-dsRNAs were not significantly different, but there was approximately 5-fold greater mortality in *D. melanogaster* larvae treated with dsRNA specific to their own species (Table 6) relative to the heterologous dsRNA treatments.

## Discussion

Feeding of dsRNA has previously been shown to induce gene silencing in a variety of terrestrial pest insects, including Lepidoptera (Turner et al. 2006; Mao et al. 2007; Bautista et al. 2009), Coleoptera (Baum et al. 2007; Whyard et al. 2009), Hymenoptera (Maori et al. 2009), Diptera (Walshe et al. 2009; Whyard et al. 2009; Li et al. 2011b), Hemiptera (Araujo et al. 2006; Shakesby et al. 2009; Whyard et al. 2009; Chen et al. 2010; Li et al. 2011a), Orthoptera (Meyering-Vos and Müller 2007), and Isoptera (Zhou et al. 2008). More recently, oral delivery of dsRNA to aquatic life stages of an insect was achieved by stabilizing the dsRNA with either the lipid transfection reagent, Effectene (Cancino-Rodezno et al. 2010), or chitosan nanoparticle microcarriers (Zhang et al. 2010). Our study, in contrast, demonstrated that dsRNA can be orally delivered to *A. aegypti* mosquito larvae simply by soaking the insects in solutions of dsRNA for as little as 2 hours. This brief dsRNA exposure was sufficient to adversely affect the development of the insects, ultimately leading to death of many of the treated individuals.

The responses to orally-delivered dsRNA in *A. aegypti* were dose-dependent, with higher concentrations of β*-tub*-dsRNA causing higher larval mortality. The highest concentration of dsRNA tested in this study was 0.5 μg/μl, but it is possible that greater mortalities may have been observed if higher doses, or longer exposures, of dsRNA were used. However, in previous experiments with feeding dsRNA to *D. melanogaster* and three other non-dipteran insect species, doses higher than 0.5 μg/μl were no more effective at inducing RNAi and killing the target insects (Whyard et al. 2009). Several other studies have similarly observed that increasing the concentration beyond an optimal dose does not improve the extent of RNAi, although the optimal concentration may vary for the species, mode of delivery, and the life stage targeted (Meyering-Vos and Müller 2007; Shakesby et al. 2009; Huvenne and Smagghe 2010). While RNAi-mediated knockdown of the targeted genes in *A. aegypti* in this study was incomplete, it was comparable to gene silencing levels observed in many other insects that have been fed dsRNA, where the extent of RNAi-induced silencing typically ranged between 40 and 60% in insects fed single doses or multiple doses of dsRNA (Araujo et al. 2006; Zhou et al. 2008; Bautista et al. 2009; Walshe et al. 2009; Zhang et al. 2010; Li et al. 2011a). The fact that there was no observed benefit to using liposome carriers in this study may be a reflection of both the sufficiently high dose of dsRNA used on highly susceptible first instar larvae, and on the sensitivity of the gene to any RNAi-induced perturbations. If lower doses of dsRNA are used, or the targeted gene is not expressed at precisely the time of dsRNA delivery, it might be useful to use transfection reagents to either stabilize the dsRNA or deliver a larger or more sustained dose to the target cells.

With this soaking method, the dsRNA did not appear to enter the insects by any route other than through the gut, as there was no measurable RNAi in individuals that failed to ingest dsRNA. RNAi was not however limited to the gut, but spread to adjacent tissues. Following soaking of the larvae in β*-tub-*dsRNA, this gene's expression in the gut was diminished 50% in the gut and 40% in the remaining carcass, suggesting that a considerable amount of the silencing dsRNA had spread beyond the gut to other tissues. For those insects treated with *hsp83*-dsRNA, reduction of this chaperone protein's transcript rendered them less tolerant to a heat stress. Although it was not determined which specific tissues exhibited RNAi-mediated knockdown of the *hsp83* transcripts, the RNAi effect was not likely limited to just the gut. Given that a significant number of larvae died from the brief heat stress following the knockdown of *hsp83* expression, it seems more plausible that many other tissues were affected, resulting in widespread irreparable heat-induced damage.

Interestingly, larvae treated with dsRNA specific to either of the two chitin synthase genes, *AeCS1* and *AeCS2*, also showed measurable knockdown of their respective transcripts, but only *AeCS1*-dsRNA adversely affected larval growth or survival. *AeCS1* shares a high level of identity (74 and 83%, respectively) with *D. melanogaster's CS-1* gene (also known as *kkv*, GenBank accession: NM_079509) and *An. gambiae's AgCHS1* gene, and is most likely the homolog of these genes, which encode the chitin synthase found in the insect epidermis. The qRT-PCR results support this conclusion, given that *AeCS1* was expressed in the carcass of the dissected larvae and not in the midgut. In contrast, *AeCS2* was expressed only in the midgut tissues, with no appreciable expression in the carcass, and is likely the homolog of *CS-2* (GenBank accession: NM_079485) and *AgCHS2* in *D. melanogaster* and *An. gambiae*, respectively. This chitin synthase gene is expressed in insect midgut cells and produces the peritrophic matrix (Merzendorfer 2006). As previously noted, the silencing of *AeCS2* did not affect the viability of the mosquito larvae, but reduction of this chitin synthesis in the gut rendered the *An. gambiae* larvae more susceptible to gut-acting insecticides like diflubenzuron (Zhang et al. 2010).

In this study and another (Zhang et al. 2010), it was observed that ingested dsRNA in mosquitoes is capable of spreading beyond the gut to other tissues. However, the mechanisms by which the dsRNA is transported from cell to cell to induce systemic RNAi are not yet elucidated. SID-1 is a cell surface dsRNA transport protein that was first identified in the nematode *Caenorhabditis elegans* (Winston et al. 2002). While *sid-1* homologs have been identified within the genomes of many eukaryotes, it appears to be curiously absent in Diptera (reviewed in Gordon and Waterhouse 2007; Tomoyasu et al. 2008; Huvenne and Smagghe 2010). Despite the absence of SID-1 in *D. melanogaster, Drosophila* S2 cells are capable of dsRNA uptake. A variety of components involved in receptor-mediated endocytic pathways that may play roles in dsRNA uptake in Diptera, and perhaps many other species, have been identified (Saleh et al. 2006). Two scavenger receptors, SC-R1 and EATER, normally involved in phagocytosis of bacterial pathogens, may also aid dsRNA uptake in cells (Ulvila et al. 2006). These receptors display specificity for multiple ligands, and dsRNA may be a previously unrecognized ligand for these receptors. It will be interesting to determine whether some of these same proteins facilitate dsRNA in mosquito gut cells, and to assess whether different dipteran species share the same mechanisms. Previous studies (Whyard et al. 2009; Zhang et al. 2010) suggested that either a transfection reagent or RNA carrier was required to deliver dsRNA to *An. gambiae* and several *Drosophila* species, respectively, and yet in our study, no transfection reagent was required to deliver dsRNA to *A. aegypti* gut cells. The apparent difference in dsRNA uptake in the two mosquitoes may reflect differences in receptor quantity or quality, or may reflect differences in the microenvironment of the gut of the two mosquito species.

While the mechanisms by which the dsRNA enters and distributes itself throughout an insect have not been identified, the fact that ingested dsRNA can induce RNAi in insects offers some intriguing possible applications. At the very least, an oral dsRNA delivery method in mosquito larvae that requires no transfection reagents to stabilize the RNA will facilitate the development of higher throughput RNAi screens in *A. aegypti*. Even with its genome fully sequenced, the majority of *A. aegypti's* genes have no confirmed function. Using a simple dsRNA soaking method, it may be possible to examine large numbers of genes’ functions by creating RNAi-mediated loss-of-function mutants, at least in larvae, where RNAi was observed to persist for at least several days post-treatment. Such highthroughput screens may identify new targets for future RNAi-based control technologies. It is unknown whether other aquatic insects can take up dsRNA as easily as *A. aegypti* larvae, but it is worth determining whether simple soaking methods can work for other invertebrates.

It is clear from our study that the delivery of certain dsRNAs could serve as mosquito larvicides. It was observed that even for a highly conserved gene such as *β-tubulin*, it is possible to design a dsRNA that can kill one species (*A. aegypti*) but not adversely affect another (*D. melanogaster*). Given that species are defined by the uniqueness of their gene sequences, it is theoretically possible to develop species-limited dsRNAs that selectively inhibit target genes in only one or a few target species. By targeting portions of genes unique to one species, dsRNAs have been designed that can kill one, but not other closely related species (Baum et al. 2007; Whyard et al. 2009). For plant-feeding insects, transgenic plants can provide the insecticidal dsRNA to pest insects in a stable and potent form (Baum et al. 2007; Mao et al. 2007), but for pest insects like mosquitoes, the dsRNA could potentially be applied to aquatic environments in formulations similar to those used for sustained release in water (reviewed in Lacey 2007). In this type of application, it will be necessary to mass-produce dsRNAs, which may be achievable by using engineered microorganisms as biofactories to synthesize the dsRNA *in vivo*.

If dsRNAs are to be harnessed as potential pesticides, it will also be important to assess the likelihood of cross-reactivity with other species. As more genomes are sequenced, this process will become easier using bioinformatic tools, but for now, the development of dsRNA-based pesticides will need to rely on identifying insect-specific genes, coupled with judicious testing of key, non-target species found in the same environments as the pest insect to be targeted. Many of our current chemical pesticides are broad-spectrum, adversely affecting many non-target species, and with growing concerns about declines in species diversity, dsRNA-based pesticides may offer some safer alternatives.

**Table 1.**
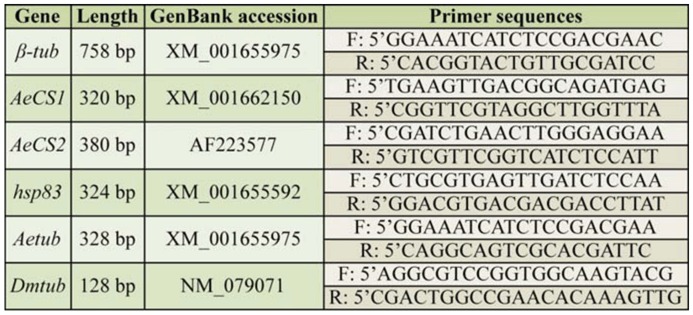
PCR Primers used to amplify genes used for dsRNA preparation.

**Table 2.**
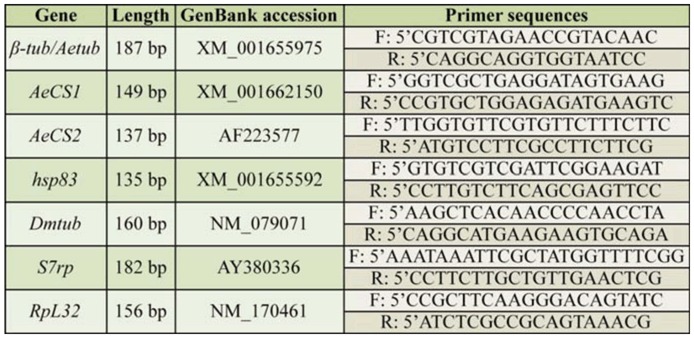
Quantitative RT-PCR primers used for determining gene knockdown.

**Table 3.**
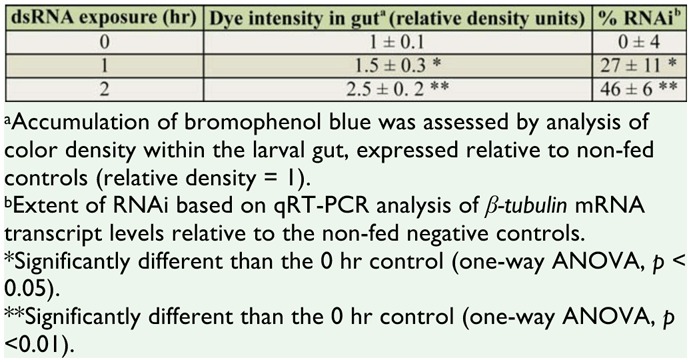
Extent of ingested *β-tubulin* -dsRNA/bromophenol dye in the gut and correlated RNAi in first instar *Aedes aegypti* larvae. Values represent the means and standard errors from 3 replicates of 10–20 larvae.

**Table 4.**
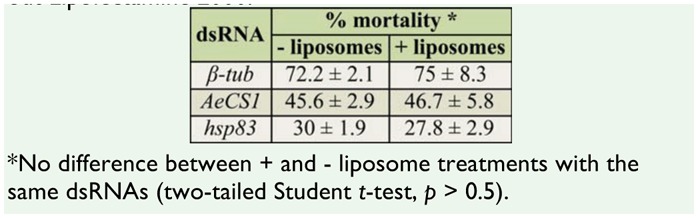
Mortality of larvae after dsRNA treatment with or with-out Lipofectamine 2000.

**Table 5.**
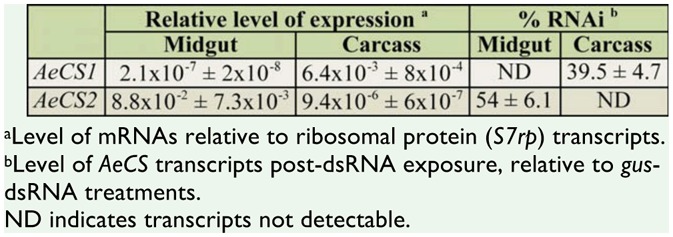
Tissue localization of the two *chitin synthase* transcripts and their knockdown following dsRNA treatments in *Aedes aegypti*. Values represent means and standard errors from 3 replicates of 5 pooled dissected tissues.

**Table 6.**
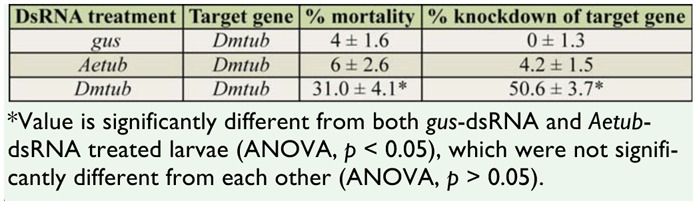
Mortality and qRT-PCR data for *Drosophila melanogaster* larvae treated with *β-tubulin* dsRNAs. The values represent the means and standard errors for 3 replicate experiments.

## Abbreviations

β-**tub**: *β-tubulin*;
AeCS: *Aedes aegypti* chitin synthase;
*Aetub*: *A. aegypti β-tub* gene;
AgCHS: *Anopheles gambiae* chitin synthase;
*Dmtub*: *D. melanogaster β-tubulin*;
dsRNA: double-stranded RNA;
*gus*: *β-glucuronidase;*
*hsp83*: *heat shock protein 83*;
RNAi: RNA interference
